# 

**Published:** 1854-11

**Authors:** 


### CONTENTS OF NO. I., VOL. II.

### ORIGINAL COMMUNICATIONS.

Notes on Anatomy—By Prof. J. C. Hughes, M. D. . 1
Effects of Medicine on the Human System—By E. C. Atkinson, M. D. . . . . . . . . 8
Labor complicated with Monstrosity—By Dr. J. Q. Egelston, . . . . . . . . . 12
Iodine in Continued Fever—By Dr. G. W. Hall, . . 14
Cholera in Boston, Mass.—By E. B. Moore, M. D. . 16
Gutta Percha Splints—By Samuel H. Case, M. D. . . 18
Case in Surgery—By Prof. J. C. Hughes, M. D. . . 20

### DEPARTMENT OF SELECTIONS.

Mental Derangement—By Roger G. Perkins, M. D. . . 22
Clinical Lecture on Whooping Cough—By R. Todd, M. D. 25
Questionable Utility of Chloroform—By Dr. Robert Lee, M. D., F. R. S. . . . . . . . . 34
Luxation of Metatarsal Bones under the Tarsus—By Mr. Tuffnell, . . . . . . . . . 37
Adhesive Plaster in the Fracture of Patella—By Dr. Neill, . . . . . . . . . . . 39
From Report to State Medical Society—By Prof. D. L. McGugin, M. D. . . . . . . . . 40
Pathological Importance of Ulceration of Os Uteri—By Charles West, M. D. . . . . . . 41
Urea in its relations to Animal Physiology—By Thos. Bischoff, . . . . . . . . . . 53
Recumbent Position during Syncope, . . . . . . 58

### EDITORIAL DEPARTMENT.

The Journal, . . . . . . . . . . 60
Medical Fogieism, . . . . . . . . . . 64
To our Readers, . . . . . . . . . . 71
Next College Session, . . . . . . . . . 72

### REVIEWS AND BIBLIOGRAPHICAL NOTICES.

Auscultation and Percussion—By Joseph Skoda, . . 74
Hughes' Clinical Introduction to the Practice of Auscultation, . . . . . . . . . . 78
A Universal Formulary—By Egglesfield Griffith, M. D. . 78
The Science and the Art of Surgery—By Prof. John Ericksen, M. D. . . . . . . . . . 79
American Journal of Pharmacy—By Prof. W. Proctor, Phil'a, 80

